# *Staphylococcus aureus* activates NRLP3-dependent IL-1β secretion from human conjunctival goblet cells using α toxin and toll-like receptors 2 and 1

**DOI:** 10.3389/fcimb.2023.1265471

**Published:** 2023-11-27

**Authors:** Dayu Li, Robin Hodges, Maria AukrustNaqvi, Jeffrey Bair, Paulo J. M. Bispo, Michael S. Gilmore, Meredith Gregory-Ksander, Darlene A. Dartt

**Affiliations:** ^1^ Schepens Eye Research Institute/Massachusetts Eye and Ear, Boston, MA, United States; ^2^ Department of Ophthalmology Harvard Medical School, Boston, MA, United States; ^3^ Department of Life Sciences and Health Faculty of Health Sciences Oslo Metropolitan University, Oslo, Norway; ^4^ Massachusetts Eye and Ear, Boston, MA, United States

**Keywords:** bacteria, conjunctiva, goblet cells, NLRP3, toll-like receptors

## Abstract

We used cultured human conjunctival goblet cells to determine (i) whether the toxigenic *S. aureus-* induced activation of the epithelial goblet cells requires two signals to activate the NLRP3 inflammasome, (ii) if one signal is mediated by TLR1, TLR2, or TLR6, and (iii) if the *S. aureus* toxin α toxin is another signal for the activation of the inflammasome and secretion of mature IL-1β. Cultured cells were incubated with siRNA to knock down the different TLRs. After stimulation with toxigenic *S. aureus* RN6390, pro-IL-1β synthesis, caspase-1 activity, and mature IL-1β secretion were measured. In a separate set of experiments, the cells were incubated with toxigenic *S. aureus* RN6390 or mutant *S. aureus* ALC837 that does not express α toxin with or without exogenous α toxin. A gentamicin protection assay was used to determine if intracellular bacteria were active. We conclude that α toxin from toxigenic *S. aureus* triggers two separate mechanisms required for the activation of the NLRP3 inflammasome and secretion of mature IL-1β. In the first mechanism, α toxin secreted from internalized *S. aureus* produces a pore, allowing the internalized bacteria and associated pathogen-associated molecular patterns to interact with intracellular TLR2 and, to a lesser extent, TLR1. In the second mechanism, α toxin forms a pore in the plasma membrane, leading to an efflux of cytosolic K^+^ and influx of Ca^2+^. We conclude that α toxin by these two different mechanisms triggers the synthesis of pro-IL-1β and NLRP3 components, activation of capase-1, and secretion of mature IL-1β to defend against bacterial infection.

## 1 Introduction

The epithelial cells of the skin and wet mucosae (gastrointestinal tract, lung, eye, and genitourinary tract) are the first line of defense against the external environment, especially from pathogens. Nod-like receptors (NLRs) are cytosolic pattern recognition receptors (PRR) that form multi-protein complexes, known as inflammasomes, in response to pathogens. Of the multiple NLRs, NLRP3 is most widely studied and has been primarily investigated in macrophages (Sellin et al., 2015). The activation of NLRP3 inflammasomes in macrophages contributes to the maintenance of health and drives numerous inflammatory diseases.

The activation of NLRP3 includes the recruitment of ASC (apoptosis-associated speck-like protein containing a caspase recruitment domain or CARD) and caspase-1, activation of caspase-1, processing and release of the pro-inflammatory cytokines interleukin (IL)-1β and IL-18, and usually pyroptotic cell death. The mechanisms that regulate the synthesis, association, and activation of the components of the NLRP3 complex are still controversial (Elliott and Sutterwala, 2015). There are multiple hypotheses that propose mechanisms for the activation of the NLRP3 inflammasome, depending upon the stimulus. NLRP3 can be activated by extracellular or intracellular mechanisms, including destabilization of lysosomes and endoplasmic reticulum (ER) stress with activation of reactive oxygen species (ROS) in mitochondria (Elliott and Sutterwala, 2015). For the action of pathogens, the two signal or priming hypothesis is the most relevant. The first signal of this hypothesis consists of extracellular pathogens activating Toll-like receptors (TLRs) such as TLR1, TLR 2, TLR 4, and TLR 6. TLR2 is known to be activated by the gram-positive bacteria *Staphylococcus aureus* (*S. aureus*) (Yoshimura et al., 1999); however, TLR2 can also form heterodimers with TLR1 and TLR6 (Wyllie et al., 2000; Triantafilou et al., 2006). A previous work shows that TLR1, TLR2, and TLR6 are expressed in the conjunctival epithelium (Ueta et al., 2004; Gornik et al., 2011), but their presence in goblet cells has yet to be addressed. The activation of TLRs stimulate a MYD88-, NFκB-, and MEK-dependent pathway that leads to increased synthesis and expression of pro-IL-1β and components of the NLRP3 inflammasome such as NLRP3, ASC, and caspase-1 (Franchi et al., 2009; Kelley et al., 2019). The second signal is the formation of a pore in the plasma membrane caused by either ATP activation of the purinergic P2X7 receptor or by bacterial pore-forming toxins such as α toxin (also known as α hemolysin). The pore opening leads to an efflux of cytosolic K^+^ and influx of extracellular Ca^2+^ that are proposed to activate caspase-1. The activation of caspase-1 cleaves pro-IL-1β, forming mature IL-1β that is secreted into the external medium (Elliott and Sutterwala, 2015) (Lamkanfi and Dixit, 2014; Sharma and Kanneganti, 2016). The secreted mature IL-1β is thought to initiate the innate-mediated inflammatory response, characterized by an infiltration of neutrophils that work to remove the bacteria and/or kill the infected epithelial cells in order to eradicate the infection.

The mechanism of NLRP3 activation and its role in bacterial infection have not been well investigated in epithelial cells. The epithelial cells of the ocular surface, which includes the cornea and conjunctiva, are key components of the innate immune system designed to defend the eye against external pathogens. We focused herein on the epithelial conjunctival goblet cells, as the conjunctiva compared to the cornea is free to robustly respond to bacteria in the tear film as it does not need to maintain transparency. In addition, the goblet cells of the conjunctiva, compared to the stratified squamous cells, can respond to bacterial infection by secretion of the protective high-molecular-weight mucin MUC5AC. Finally, goblet cells from the colon were recently recognized as major immune cells with secreted mucus playing a critical role (Johansson et al., 2014). We used rat and human conjunctival goblet cells to study the role of NLRP3 in bacterial conjunctivitis and focused on the effect of *S. aureus*. We found that the components of the NLRP3 inflammasome, including NLRP3, ASC, and caspase-1, were constitutively expressed in rat and human conjunctival goblet cells *in vivo* and *in vitro* under basal conditions (McGilligan et al., 2013). Toxigenic *S. aureus* RN6390 activated the NLRP3 inflammasome via the caspase-1 pathway and stimulated the secretion of mature IL-1β, an indicator of inflammasome activation. These results demonstrated that conjunctival goblet cells contribute to the innate immune response of the ocular surface.

The goal of the present study was to, first, determine whether toxigenic *S. aureus-*induced activation of the epithelial goblet cells requires two signals to activate the NLRP3 inflammasome. Second, we investigated if one of these signals is mediated by TLR1, TLR2, or TLR6. Lastly, we explored if the *S. aureus* α toxin is the other signal required for the activation of the inflammasome and secretion of mature IL-1β. We used siRNA to knock down the different TLRs in primary cultures of human conjunctival goblet cells along with mutant bacteria lacking α toxin to determine if both TLR activation and α toxin-induced pore formation are required for inflammasome activation in conjunctival goblet cells.

## 2 Materials and methods

### 2.1 Human tissue

Conjunctival tissue was obtained from cadavers by the Heartland Lions Eye Bank (Kansas, MO, USA) and Eversight Eye Bank (Ann Arbor, MI, USA). The Massachusetts Eye and Ear IRB deemed that this did not constitute use of human tissue and thus was exempt from official IRB review. The tissue was placed in Optisol medium within 18 h of death. The conjunctiva was cleaned of connective tissue before use.

### 2.2 Cell culture

Goblet cells were grown in organ culture from human conjunctiva as described previously (Shatos et al., 2003). Briefly, pieces of minced tissue were placed in RPMI 1640 medium supplemented with 10% fetal bovine serum (FBS), 2 mM glutamine (Lonza, Walkersville, MD, USA), and 100 mg/ml penicillin/streptomycin in six-well plates. After the nodules of cells appeared, the tissue plug and non-goblet cells were removed. After 7 days, the goblet cells were trypsinized and plated in 6-, 24-, or 96-well plates. The cultured cells were identified by multiple well-defined criteria (Dartt et al., 2011; Li et al., 2012; Li et al., 2013a; Li et al., 2013b; McGilligan et al., 2013).

### 2.3 Bacterial culture and challenge of human conjunctival goblet cells

Two different strains of bacteria were used in the present study. As a representative toxigenic strain, *S. aureus* RN6390 that contains the key global regulators Sar and Agr and produces α, β, and γ toxins (Peng et al., 1988) and *S. aureus* strain ALC837, which is genetically identical except for the mutation of the α toxin gene (Chien et al., 1999), were used. The bacteria were grown at 37°C to an OD595 nm of 0.5 (early log phase) and suspended in fresh RPMI-1640 medium with 1% FBS.

First-passage human conjunctival goblet cells were seeded in culture plates in medium without antibiotics at 24 h prior to infection. The bacteria were added at a multiplicity of infection (MOI) of 20 or, in one experiment 200 as indicated and incubated for 4 h at 37°C in 5% CO_2_.

### 2.4 Depletion of TLR1, TLR2, and TLR6 proteins

First-passage human goblet cells were cultured in RPMI 1640 with 10% FBS. Predesigned TLR1, TLR2, and TLR6 siRNAs were obtained from Thermo Scientific Dharmacon RNAi Technologies (Lafayette, CO, USA). The transfection of siRNA was performed with the DharmaFECT 1 siRNA Transfection Reagent following the manufacturer’s protocol. The siRNA constructs were added at a final concentration of 50 nM for TLR1 and TLR2 siRNA and 100 nM for TLR6 siRNA in antibiotic-free RPMI 1640 as described previously (Li et al., 2013a). Scrambled siRNA (sc-siRNA, 100 nM) was used as a negative control. The cells transfected with siRNA were cultured for 48–72 h before the experimental analysis. The amount of TLR2 and TLR6 after incubation with siRNA was determined by Western blotting analysis. The amount of TLR1 was analyzed by qPCR.

### 2.5 Western blotting analysis of amounts of TLR2, TLR6, and pro-IL1β protein

First-passage human conjunctival goblet cells were seeded in six-well plates. After incubation with bacteria, the supernatant was removed, and the cells were lysed in RIPA buffer. For depletion experiments, the cells were incubated with no additions, sc-siRNA, or target siRNA at different concentrations for 48-72 h. Protein was collected for analysis by Western blotting. The primary antibodies for Western blotting analysis were used at the following dilutions: 1:400 TLR2, 1:500 TLR6, 1:400 pro-IL1β, and 1:1,000 β-actin. The pro-IL-1β antibody was purchased from R&D Systems (Minneapolis, MN, USA), the TLR2 antibody was from Novus Biochemicals (Littleton, CO, USA), the TLR6 antibody was from Thermo Fisher Scientific (Waltham, MA, USA), and the β-actin antibody was from Sigma Aldrich (St. Louis, MO, USA). The pro-IL-1β amount was normalized to β-actin. For detection, the secondary antibodies used were donkey anti-sheep (R&D Systems) and chicken anti-goat (EMD Millipore, Billerica, MA, USA) conjugated to horseradish peroxidase. Immunoreactive bands were visualized by using the enhanced chemiluminescence method (Thermo Scientific). The results were expressed as fold change compared to the normalized basal level that was set to 1.

### 2.6 qPCR for analysis of TLR1 expression

Total RNA was isolated from siRNA-transfected human goblet cells with TRIzol (Ambion, Austin, TX) according to the manufacturer’s instructions. Contaminating DNA was removed using TURBO DNA-free Kit (Ambion, Austin, TX, USA). Further RNA cleanup was done using RNeasy MinElute Cleanup Kit (Qiagen Inc., Valencia, CA, USA). The RNA concentration was determined using Nanodrop 2000 Spectrophotometer (Thermo Scientific, Waltham, MA, USA).

The first-strand cDNA synthesis reaction was performed with the SuperScript III first-strand synthesis system SuperMix kit (Invitrogen, Grand Island, NY, USA). The primer sequence information were as follows—TLR1 forward: ATT CCG CAG TAC TCC ATT CC, TLR1 reverse: TTT GCT TGC TCT GTC AGC TT, β-actin forward: GGA CTT CGA GCA AGA GAT GG, β-actin reverse: AGC ACT GTG TTG GCG TAC AG. The 20-μl real-time PCR amplification reactions were performed using Kapa SYBR Fast qPCR Kit (Kapa Biosystems, Woburn, MA, USA) with thermal cycling conditions of 1 cycle at 95°C for 3 min, 40 cycles at 95°C for 10 s, 58.5°C for 10 s, and 72°C for 15 s on the Eppendorf Realplex2 system (Eppendorf AG, Hamburg, Germany). Each assay was carried out in triplicate. The relative expression of mRNA was normalized to the relative expression of endogenous β-actin mRNA. The relative quantization of the TLR1 mRNA in each sample was calculated using the comparative threshold cycle method.

### 2.7 FLICA assay for active caspase-1 analysis

Active caspase-1 was detected using a fluorescent inhibitor of caspases (FAM-FLICA Caspase 1 Assay Kit, Immunochemistry Technologies, Bloomington, MN, USA) according to the manufacturer’s instructions. First-passage human goblet cells were grown in 96-well plates. Before use, the media was changed to antibiotic-free RPMI containing 1% FBS. The cells were then stimulated with bacteria for 4 h, and 10 μl of 30X FLICA solution was added. Following incubation, the cells were stained with Hoechst 33342 stain (0.5% w/v) and viewed on an inverted phase-contrast microscope equipped for epifluorescence (Eclipse TE300, Nikon, Tokyo, Japan) with UV filter and with excitation 490 nm and emission >520 nm for green fluorescence for caspase-1-positive cells and excitation 365 nm and emission 480 nm for the visualization of nuclei stained with the Hoechst dye. The total number of nuclei in four ×40 fields of view was counted, and the number of cells staining green (indicating active caspase-1) was expressed as fold increase over basal.

### 2.8 Mature IL-1β secretion

The supernatant from cells in which pro-IL-1β was measured was used to determine the amount of mature IL-1β by ELISA (R&D Systems), which was performed according to the manufacturer’s instructions. The ELISA detected IL-1β over a range of 1–2,500 pg/ml. The values were expressed as fold increase over basal.

### 2.9 Measurement of internalized bacteria and access by α toxin

Cultured conjunctival goblet cells were incubated with bacteria or buffer for 4 h followed by gentamycin (200 μg/ml gentamicin; Sigma-Aldrich) to kill extracellular bacteria or buffer for 2 h and then buffer or α toxin (3 μg/ml; Sigma Aldrich) for 30 min. The amount of pro-IL1β was measured in the cell lysate and of mature IL-1β in the supernatant as described above.

### 2.10 Gentamicin protection assay

To determine if toxigenic and non-toxigenic *S. aureus* invaded human conjunctival goblet cells, gentamicin protection assay was performed as previously described (Hess et al., 2003). Toxigenic RN6390, non-toxigenic ALC837, and non-toxigenic *S. aureus* ALC837 plus α toxin were each added at an MOI of 200 to first-passage goblet cells that had been seeded onto 24-well plates in antibiotic-free RPMI 1640 media. The bacteria and cells were incubated for 4 h. Unattached bacteria were removed by washing, and gentamicin (200 μg/ml) was added for 2 h to kill extracellular bacteria. After incubation, the cells were washed and lysed with 0.5% Triton X-100. Viable intracellular *S. aureus* in the lysate were quantified by serial dilution from 10^-1^ to 10^-3^. A 100-μl aliquot of lysate from each dilution and the undiluted lysate were plated in triplicate using a sterile glass plate spreader on BHI-agar plates and incubated overnight at 37°C. Colony-forming units (CFUs) per milliliter in the lysate for each dilution were calculated, and the average CFU/ml was determined.

In each assay, negative controls of gentamicin added to both strains of *S. aureus* in the absence of cells were included to determine the killing of extracellular bacteria. For these controls, both *S. aureus* strains were added at MOI of 1:200 to culture plates and incubated for 4 h, followed by addition of gentamicin at 200 μg/ml for 2 h. After incubation, 100-μl aliquots of media were plated on BHI-agar plates in duplicate. The CFUs/ml in the media were calculated.

### 2.11 Statistical analysis

The results are presented as mean ± SEM. Data were analyzed for significance using Student’s *t*-test. A *p*-value of <0.05 was considered statistically significant.

## 3 Results

### 3.1 Human conjunctival goblet cells require TLR2 for the *S. aureus* RN6390-induced production of pro-IL-1β, activation of caspase-1, and secretion of mature IL-1β

We determined whether human conjunctival goblet cells require TLR2 to recognize *S. aureus* and activate the NLRP3 inflammasome. TLR2 recognizes several cell wall components of *S. aureus*, including peptidoglycan, lipoteichoic acid, and lipoprotein and is essential in the priming of the NLRP3 inflammasome (Nilsen et al., 2008; Pietrocola et al., 2011; Wang et al., 2020). The Western blot analysis of protein lysates derived from primary human conjunctival goblet cells demonstrated a constitutive expression of TLR2 at the expected molecular weight of 95 kDa (**Supplementary Figure S1A**, lane 1). To determine whether the goblet cells require TLR2 for the *S. aureus*-induced activation of the NLRP3 inflammasome, we used siRNA to knock down TLR2 in cultured cells. Compared to basal levels, 50 and 100 nM TLR2 siRNA decreased the amount of TLR2 protein by 77% and 68%, respectively (**Supplementary Figure S1A**). TLR2 siRNA at 50 nM was used in the subsequent experiments. In contrast, sc-siRNA did not substantially alter the amount of TLR protein.

**Figure 1:**
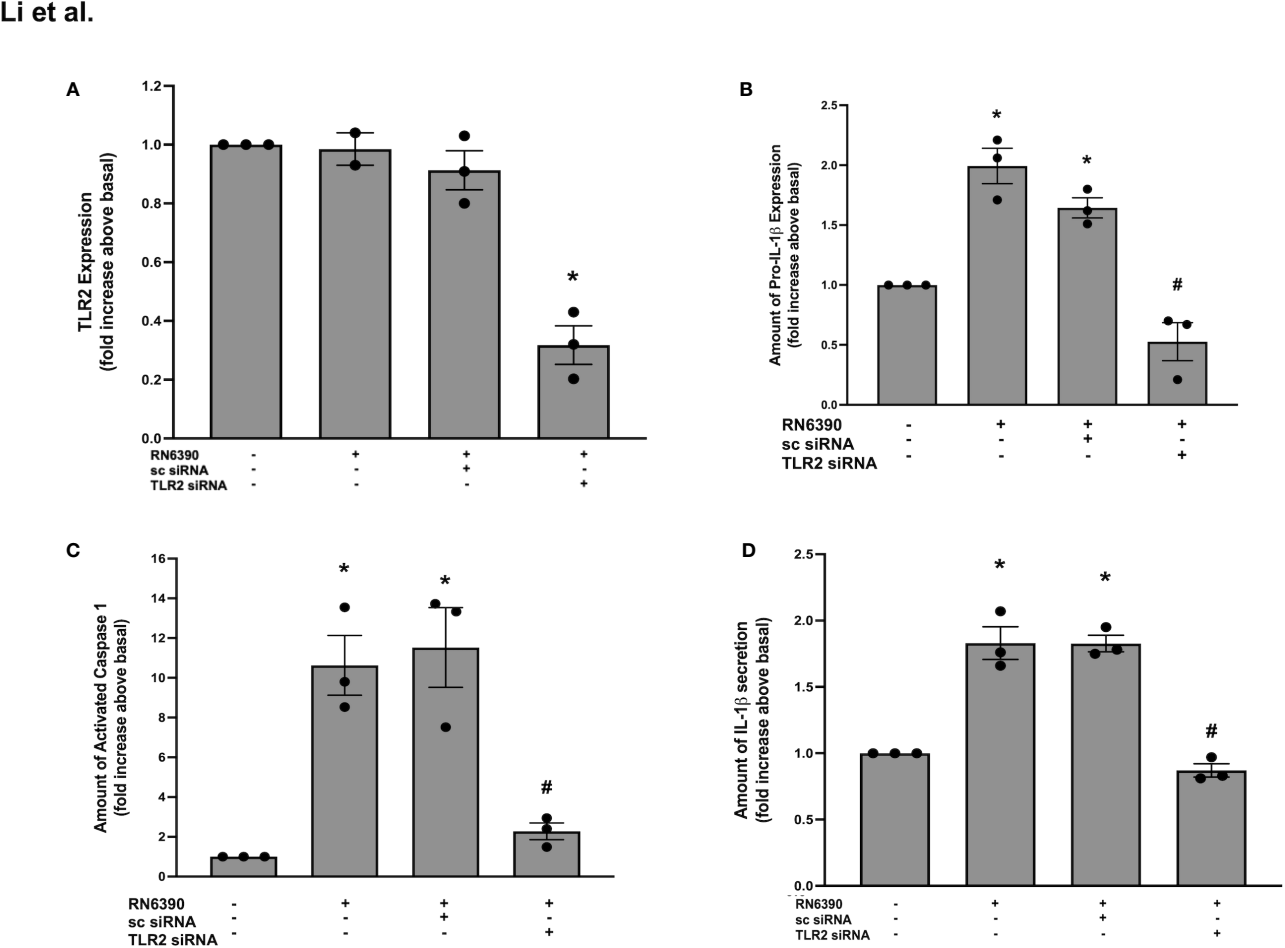
TLR2 plays a role in *S. aureus* RN6390-induced activation of NLRP3 inflammasome and production of pro-IL-1β and secretion of mature IL-1β. Cultured human conjunctival goblet cells were incubated with *S. aureus* RN6390 (multiplicity of infection 20) in the presence or absence of 50 nM TLR2 siRNA or sc-siRNA. Western blot analysis was performed for TLR2, the blots were scanned, and the amount of TLR2 relative to β-actin is plotted in **(A)**. The amount of pro-ILβ was also determined by Western blot. The blots were scanned, and the amount of pro-ILβ is shown in **(B)**. The amount of caspase 1 was determined by FLICA assay. The amount of activated caspase 1 was measured and is shown in **(C)**. The amount of mature IL-1β was determined by ELISA and shown in **(D)**. Data are mean ± SEM from three independent experiments. *, significant difference from basal; #, significance from strain RN6390 alone.

To examine the function of TLR2 in the conjunctival goblet cell response to toxigenic *S. aureus*, goblet cells were incubated with strain RN6390 in the presence or absence of TLR2 siRNA or sc-siRNA as a negative control. The Western blot analysis demonstrated that the incubation of goblet cells with *S. aureus* RN6390 did not increase the TLR2 expression above basal levels (**Supplementary Figure S1B**; **Figure 1A**). Incubation with sc-siRNA plus *S. aureus* RN6390 did not alter the response compared to *S. aureus* RN6390 alone. However, incubation with strain RN6390 plus TLR2 siRNA significantly reduced the TLR2 expression to below basal levels (0.3 ± 0.1-fold of basal). While incubation with *S. aureus* RN6390 had no effect on goblet cell expression of TLR2, it significantly increased the levels of pro-IL-1β production by 2.0 ± 0.2-fold compared to basal (**Supplementary Figure S1C**; **Figure 1B**). This induction of pro-IL-1β was unaltered following pre-incubation with sc-siRNA but completely blocked when goblet cells were pre-incubated with TLR2-siRNA before the addition of *S. aureus* RN6390.

As another measure of NLRP3 inflammasome activation, caspase-1 activity was evaluated using the FLICA probe FAM-YVAD-FMK that specifically labels active caspase-1 (Tseng et al., 2013). Incubation with *S. aureus* RN6390 alone increased the activity of caspase-1 by 10.6 ± 1.5-fold of basal (**Supplementary Figure S1D**; **Figure 1C**). Pre-incubation with sc-siRNA did not alter the *S. aureus* RN6390 increase in the activity of caspase-1, while pre-incubation with TLR2-siRNA significantly and substantially blocked caspase-1 activity (2.3 ± 0.4-fold of basal).

Secretion of mature IL-1β is the final end product of NLRP3 inflammasome activation, and ELISA was used to measure the amount of IL-1β secreted into the supernatant of the above-mentioned cultures. Incubation with *S. aureus* RN6390 alone induced a significant secretion of mature IL-1β secretion (1.8 ± 0.1-fold of basal) (**Figure 1D**). Similarly to caspase-1 activity, pre-incubation with sc-siRNA did not alter, but TLR2-siRNA completely blocked, the *S. aureus*-induced secretion of mature IL-1β. Taken together, these data demonstrate that human conjunctival goblet cells require TLR2 expression for the toxigenic *S. aureus*-induced upregulation of pro-IL-1β, activation of the NLRP3 inflammasome, and secretion of mature IL-1β.

### 3.2 TLR1 participates in the *S. aureus* RN6390-induced production of pro-IL-1β and secretion of mature IL-1β

**Figure 2:**
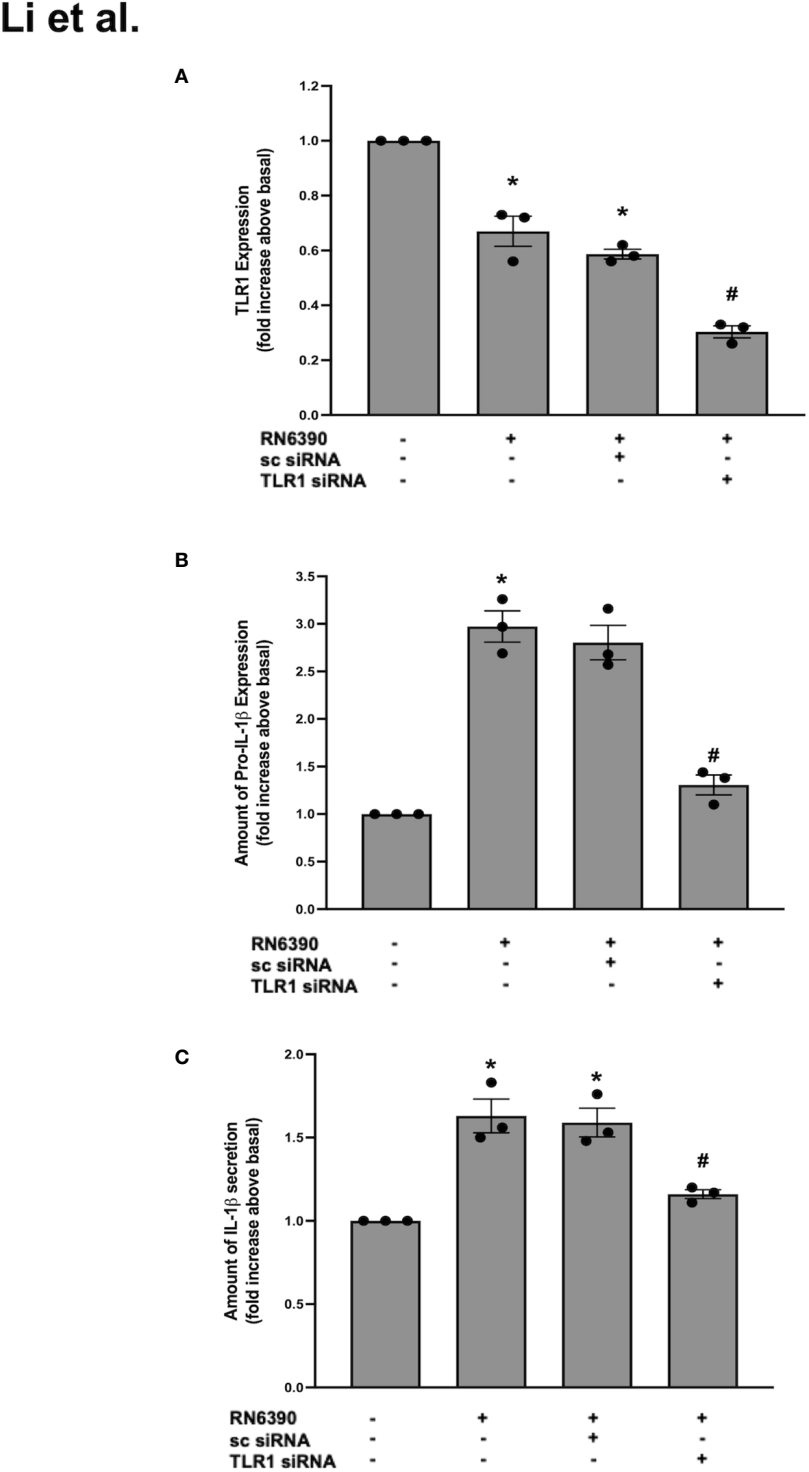
TLR1 plays a role in *S. aureus* RN6390-induced production of pro-IL-1β and secretion of mature IL-1β. Cultured human conjunctival goblet cells were incubated with *S. aureus* RN6390 (multiplicity of infection, 20) in the presence or absence of 50 nM TLR1 siRNA or sc-siRNA. Q-PCR was performed for TLR2, quantified, and the amount of TLR2 relative to β-actin is plotted in **(A)**. Western blot analysis was performed for pro-ILβ, the blots were scanned, and the expression of pro-IL-β is shown in **(B)**. The amount of secreted IL-1β was determined by ELISA and is shown in **(C)**. Data are mean ± SEM from three independent experiments. *, significant difference from basal; #, significant difference from strain RN6390.

TLR2 recognizes cell wall components of *S. aureus* and forms heterodimers with TLR1 and/or TLR6 (Wyllie et al., 2000; Triantafilou et al., 2006). To determine whether TLR1 participates in the goblet cell response to *S. aureus*, we first interrogated whether TLR1 was expressed in primary human conjunctival goblet cells. TLR1 is expressed in the conjunctival epithelium, but a specific expression in goblet cells was not yet published (Gornik et al., 2011). Using several antibodies, we were unable to detect TLR1 in primary human conjunctival goblet cells by Western blot (data not shown). However, qPCR revealed that TLR1 mRNA was constitutively expressed in primary human conjunctival goblet cells (**Figure 2A**, lane 1). Compared to basal mRNA, the levels of TLR1 were decreased following incubation with *S. aureus* alone or with sc-siRNA (**Figure 2A**, lanes 2 and 3). In contrast, pre-incubation with 50 nM TLR1 siRNA significantly reduced the mRNA levels of TLR1 below that observed with *S. aureus* alone (**Figure 2A**, lane 4).

In the same set of experiments, low levels of pro-IL-1β were detected in the goblet cells cultured under basal conditions, and the addition of *S. aureus* RN6390 significantly increased the amount of pro-IL-1β protein by 3.0 ± 0.2-fold compared to basal (**Supplementary Figure S2A**; **Figure 2B**). This increase was unaltered by sc siRNA but was significantly reduced to 1.3 ± 0.1-fold of basal by TLR1-siRNA. Similarly, incubation with *S. aureus* RN6390 alone increased the mature IL-1β secretion by 1.6 ± 0.1-fold compared to basal (**Figure 2C**). This increase was unaltered by sc siRNA but significantly reduced by 60% to 1.2 ± 0.02-fold of basal in the presence of TLR1-siRNA.

The TLR2 and TLR1 results taken together demonstrate that siRNA successfully knocked down TLR2 and TLR1 below basal levels. However, the knockdown of TLR2 decreased the *S. aureus*-induced secretion of mature IL-1β by greater than 100% compared to only a 60% reduction in goblet cells treated with TLR1 siRNA. Thus, we conclude that goblet cells require TLR2 and, to a lesser extent, TLR1 to activate the NLRP3 inflammasome in response to *S. aureus* RN6390.

### 3.3 TLR6 does not participate in the *S. aureus* RN6390-induced production of pro-IL-1β, activation of caspase-1, and secretion of mature IL-1β

As stated above, TLR2 can heterodimerize with either TLR1 or TLR6. The Western blot analysis of protein lysates derived from primary human conjunctival goblet cells demonstrate a constitutive expression of TLR6 at the expected molecular weight of 92 kDa (**Supplementary Figure S3A**). To determine whether TLR6 participates in the goblet cell response to *S. aureus*, we used siRNA to knock down TLR6 in goblet cells. The addition of 100 nM sc-siRNA caused only a 30% reduction of TLR6 compared to basal. In contrast, incubation with 25, 50, and 100 nM of TLR6 siRNA reduced the expression of TLR6 by 40%, 44%, and 80%, respectively, compared to basal. TLR6 siRNA at 100 nM was used in the subsequent experiments.

**Figure 3:**
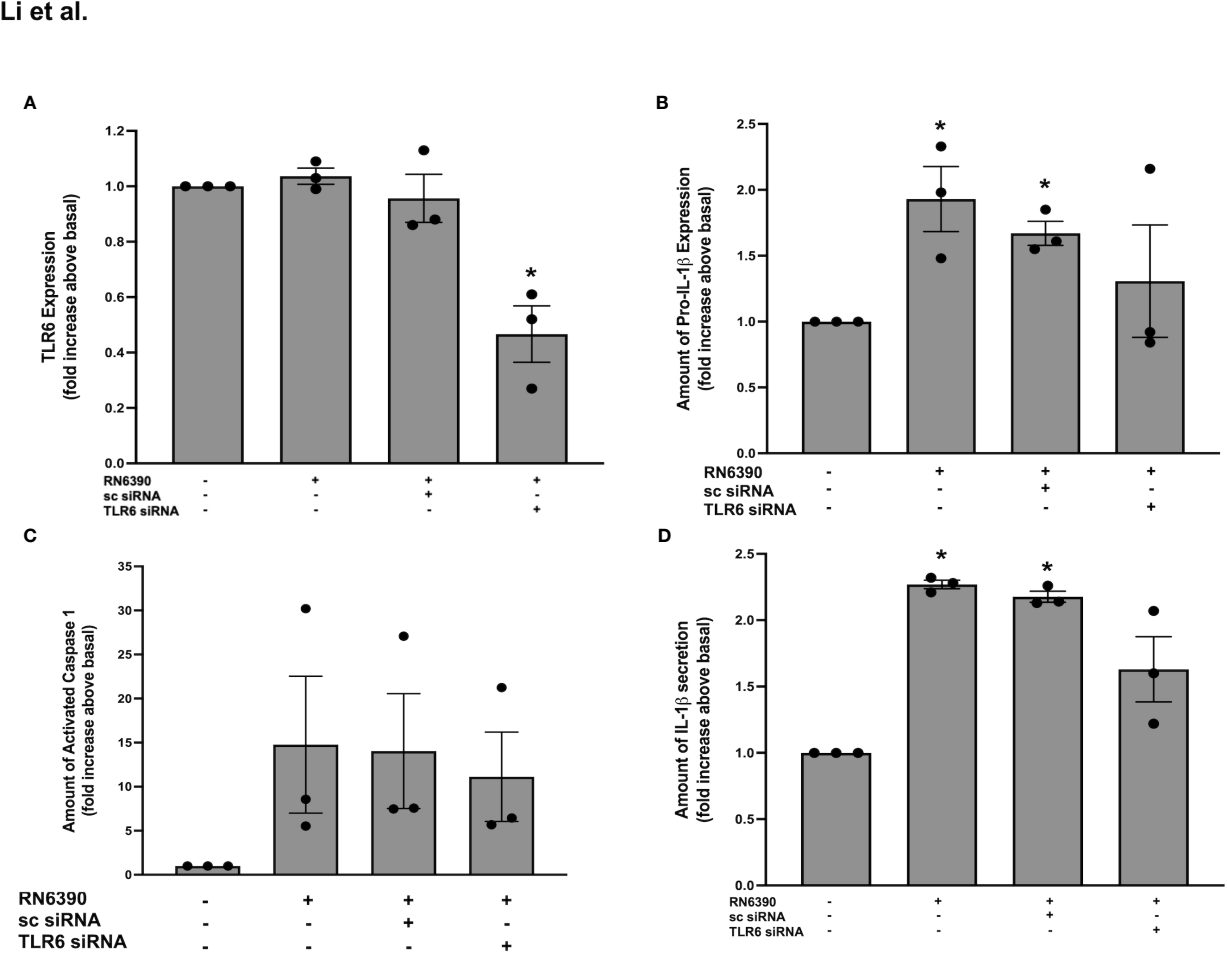
TLR6 plays a role in *S. aureus* RN6390-induced activation of NRLP3 inflammasome and production of pro-IL-1β and secretion of mature IL-1β. Cultured human conjunctival goblet cells were incubated with *S. aureus* RN6390 (multiplicity of infection, 20) for 4 h in the presence or absence of 100 nM TLR6 siRNA or sc-siRNA. Western blots were performed, the blots were scanned, and the amount of TLR6 relative to β-actin plotted in **(A)**. The amount of pro-ILβ was determined by Western blot, the blots were scanned, and the amount of pro-ILβ is shown in **(B)**. The amount of caspase 1 was determined by FLICA assay and shown in **(C)**. The amount of mature IL-1β was determined by ELISA and is shown in **(D)**. Data are mean ± SEM from three independent experiments. *, significant difference from basal.

As observed with TLR2 and TLR1, incubation with *S. aureus* RN6390 alone did not increase the expression of TLR6 on primary human conjunctival goblet cells compared to no addition (**Supplementary Figure S3B**; **Figure 3A**). Pre-incubation with TLR6 siRNA, but not sc-siRNA, significantly reduced the TLR6 expression to 0.5 ± 0.1-fold of basal. The effect of TLR6 knockdown was then tested on pro-IL-1β expression. Incubation of goblet cells with *S. aureus* RN6390 alone increased the pro-IL-1β expression by 1.9 ± 0.3-fold compared to basal (**Supplementary Figure S3C**; **Figure 3B**). Pre-incubation with either sc siRNA or TLR6 siRNA had no significant effect on the *S. aureus*-induced production of pro-IL-1β, with pro-ILβ detected at 1.3 ± 0.4-fold of basal. When caspase-1 activity was measured under the same conditions as pro-IL-1β, incubation with *S. aureus* RN6390 alone increased the activity of caspase-1 to 14.8 ± 7.8-fold over basal (**Supplementary Figure S3D**; **Figure 3C**). Pre-incubation with sc-siRNA as well as TLR6 siRNA had no inhibitory effect on *S. aureus*-induced caspase-1 activation, with caspase-1 activity found to be 11.1 ± 5.1 fold of basal with TLR6 siRNA.

Lastly, the secretion of mature IL-1β was investigated. Incubation of goblet cells with *S. aureus* RN6390 increased the secretion of mature IL-1β by 2.3 ± 0.03-fold compared to basal (**Figure 3D**). Pre-incubation sc-siRNA, as well as TLR6 siRNA, did not alter the *S. aureus*-induced secretion of mature IL-1β. These data demonstrate that TLR6 does not participate in the goblet cell response to *S. aureus* and TLR6 is not required for the *S. aureus*-induced production of pro-IL-1β, activation of caspase-1, and secretion of mature IL-1β.

Our results demonstrate that goblet cells require TLR2 to a large extent and TLR1 to a lesser extent, but not TLR6, for the *S. aureus-*induced production of pro-IL-1β as well as the activation of caspase-1 and secretion of mature IL-1β.

### 3.4 α toxin in toxigenic *S. aureus* RN6390 is needed to activate the NLRP3 inflammasome in human conjunctival goblet cells and induce the secretion of mature IL-1β

**Figure 4:**
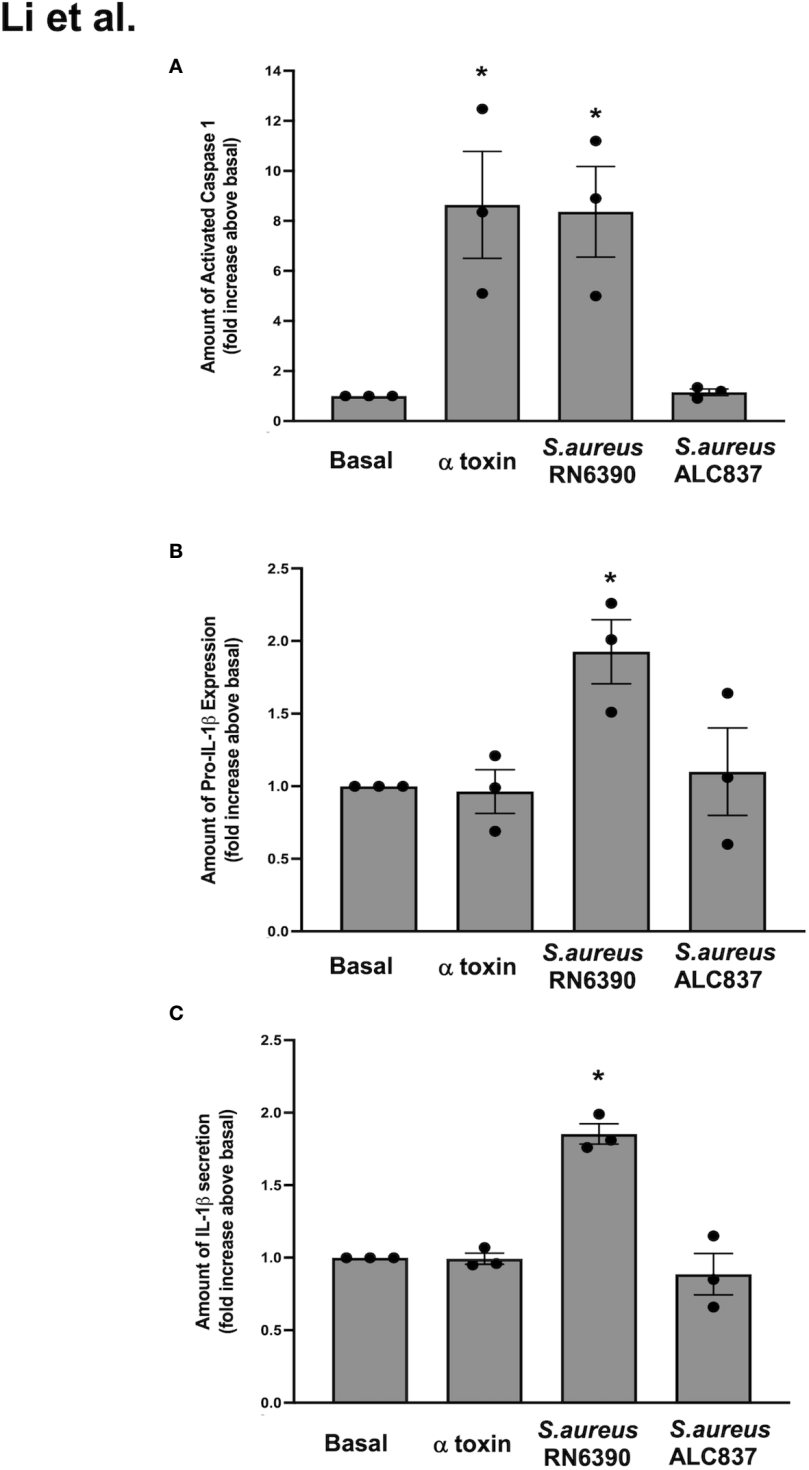
*S. aureus* RN6390 requires α toxin to activate the NLRP3 inflammasome and induce the production of pro-IL1β and secretion of mature IL-1β. Cultured human conjunctival goblet cells were treated with 1 μg/ml α toxin, *S. aureus* RN6390, or *S. aureus* ALC837 (multiplicity of infection, 20) for 4 h. The activity of caspase 1 was determined by FLICA and shown in **(A)**. The amount of pro-IL1β was measured by Western blot, the blots were scanned, and the amount of pro-IL1β is plotted in **(B)**. The amount of secreted mature IL-1β was measured by ELISA and is shown in **(C)**. Data are mean ± SEM from three independent experiments. *, significant difference from basal.

In conjunctival goblet cells, the *S. aureus*-induced activation of TLR2/1 is required for the induction of pro-IL-1β, inflammasome activation, and secretion of mature IL-1β. In addition, pore-forming toxins expressed by toxigenic *S. aureus* serve as another signal that could activate the NLRP3 inflammasome in conjunctival goblet cells. The *S. aureus*-derived α toxin was shown to replace the requirement of ATP and the P2X7 receptor to induce caspase-1 activation through NLRP3 (Munoz-Planillo et al., 2009). To determine the ability of α toxin to activate the NLRP3 inflammasome in human conjunctival goblet cells, we first incubated goblet cells with α toxin alone. The ability to activate the NLRP3 inflammasome was determined by measuring the caspase-1 activity. α toxin alone induced caspase-1 activation by 8.6 ± 2.1-fold over basal and was equal to the level of caspase-1 activation induced by *S. aureus* RN6390 (**Figure 4A**). This result indicates that α toxin is a key mediator of the *S. aureus*-induced activation of the NLRP3 inflammasome. To confirm that α toxin is needed for the *S. aureus*-induced activation of the NLRP3 inflammasome, we used a mutant *S. aureus* ALC837 that is identical to strain RN6390, except that α toxin is not produced. The mutant *S. aureus* ALC837 infection was unable to induce caspase-1 activation under identical conditions, demonstrating that α toxin is needed for the *S. aureus-*induced activation of the NLRP3 inflammasome.

We next evaluated the effect of α toxin, *S. aureus* RN6390, and *S. aureus* ALC837 on pro-IL-1β expression. α toxin alone had no effect on the levels of pro-IL-1β protein compared to basal (**Supplementary Figure S4**; **Figure 4B**), while *S. aureus* RN6390 significantly increased pro-IL-1β protein expression by 1.9 ± 0.2-fold compared to basal. In contrast, *S. aureus* ALC837 did not increase the pro-IL-1β protein expression over basal levels. Similar effects were observed when measuring the secretion of mature IL-1β in that α toxin alone did not increase the secretion of mature IL-1β, while *S. aureus* RN6390 significantly increased it by 1.9 ± 0.1-fold and *S. aureus* ALC837 had no effect (**Figure 4C**).

**Figure 5:**
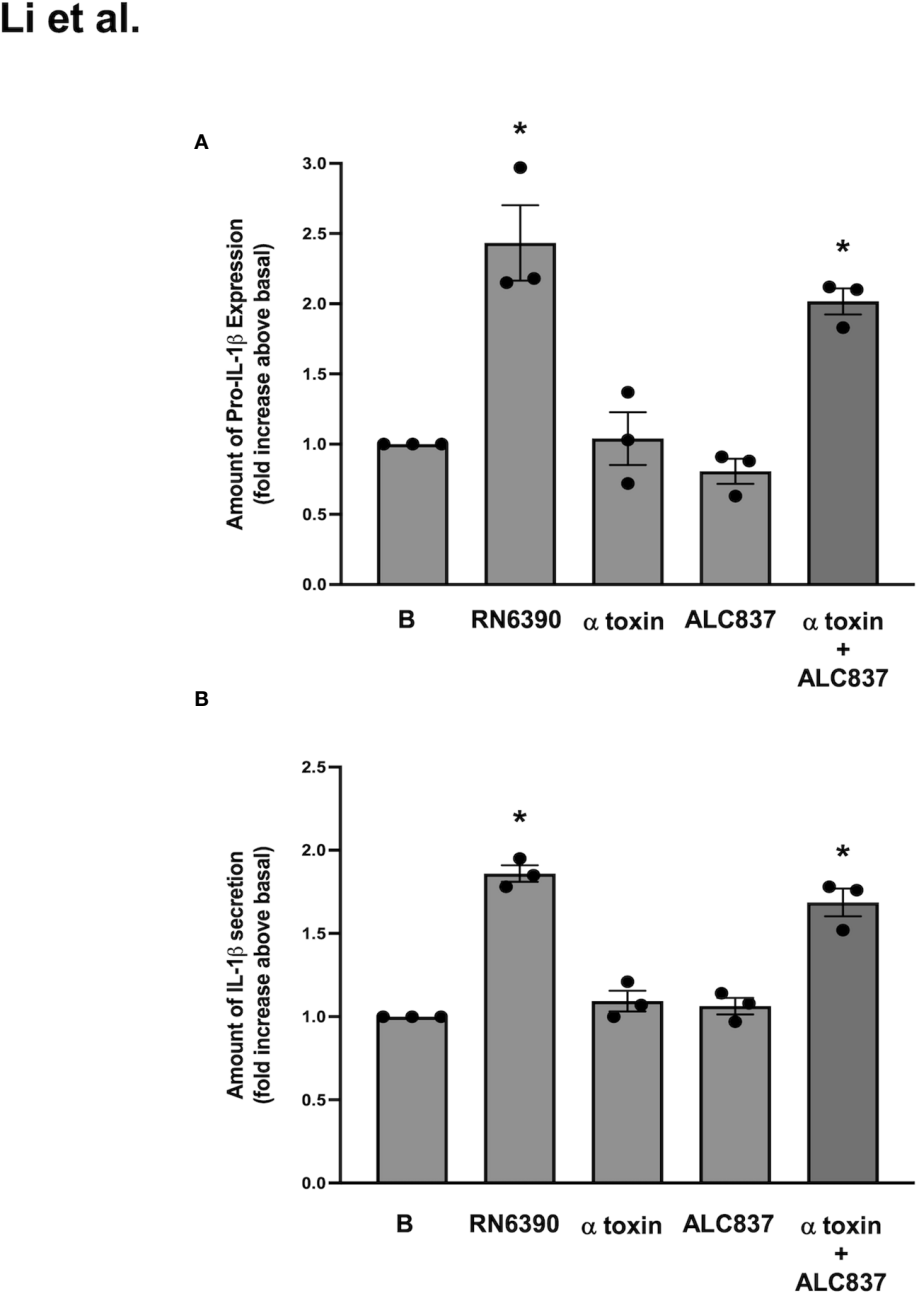
*S. aureus* ALC837 needs α toxin to induce the production of pro-IL1β and secretion of mature IL-1β. Cultured human conjunctival goblet cells were treated with *S. aureus* ALC837 (multiplicity of infection, 20), 1 μg/ml α toxin, and *S. aureus* RN6390 or ALC837 plus α toxin for 4 h. The amount of pro-IL1β was measured by Western blot, the blots were scanned, and the amount of pro-IL1β is plotted in **(A)**. The amount of secreted mature IL-1β was measured by ELISA and shown in **(B)**. Data is mean ± SEM from three independent experiments. *, significant difference from basal.

Reconstitution or add-back experiments were next performed to determine if α toxin and *S. aureus* ALC837 added together induced the same response as *S. aureus* RN6390 alone. The expression of pro-IL-1β and the secretion of mature IL-1β were measured as end points. *S. aureus* RN6390 significantly increased the expression of pro-IL-1β protein by 2.4 ± 0.3-fold compared to basal (**Supplementary Figure S5**; **Figure 5A**), while neither α toxin alone nor *S. aureus* ALC837 alone increased the expression of pro-IL-1β protein compared to basal. The simultaneous addition of α toxin and *S. aureus* ALC837 induced pro-IL-1β protein expression to a level equivalent to that induced by *S. aureus* RN6390. Similar results were obtained when the secretion of mature IL-1 β was measured. *S. aureus* RN6390 significantly increased the expression of mature IL-1β secretion by 1.9 ± 0.05-fold compared to basal (**Figure 5B**). Neither α toxin alone nor *S. aureus* ALC837 alone increased the secretion of mature IL-1β compared to basal, while the simultaneous addition of α toxin and *S. aureus* ALC837 stimulated the pro-IL-1β expression to a level comparable to the *S. aureus* RN6390 response. These studies together demonstrate that α toxin is needed for the *S. aureus*-induced activation of the NLRP3 inflammasome.

### 3.5 *S. aureus* RN6390 and its α toxin mutant ALC837 are internalized by human conjunctival goblet cells

In **Figure 5**, we show that the incubation of conjunctival goblet cells with mutant *S. aureus* ALC837 alone does not trigger the production of pro-IL-1β until α toxin is added. Because this *S. aureus* mutant is only lacking α toxin, it should still be able to bind to cell-surface TLR2, activate the MYD88 and NFκB-dependent pathway, and stimulate the synthesis of pro-IL-1β. The results in **Figure 5A**, however, show that, in the absence of α toxin, the mutant *S. aureus* is unable to induce the synthesis of pro-IL-1β. We thus asked if the activation of TLR2 and subsequent inflammasome activation was dependent upon bacterial internalization and presence of the pore-forming α toxin.

**Figure 6:**
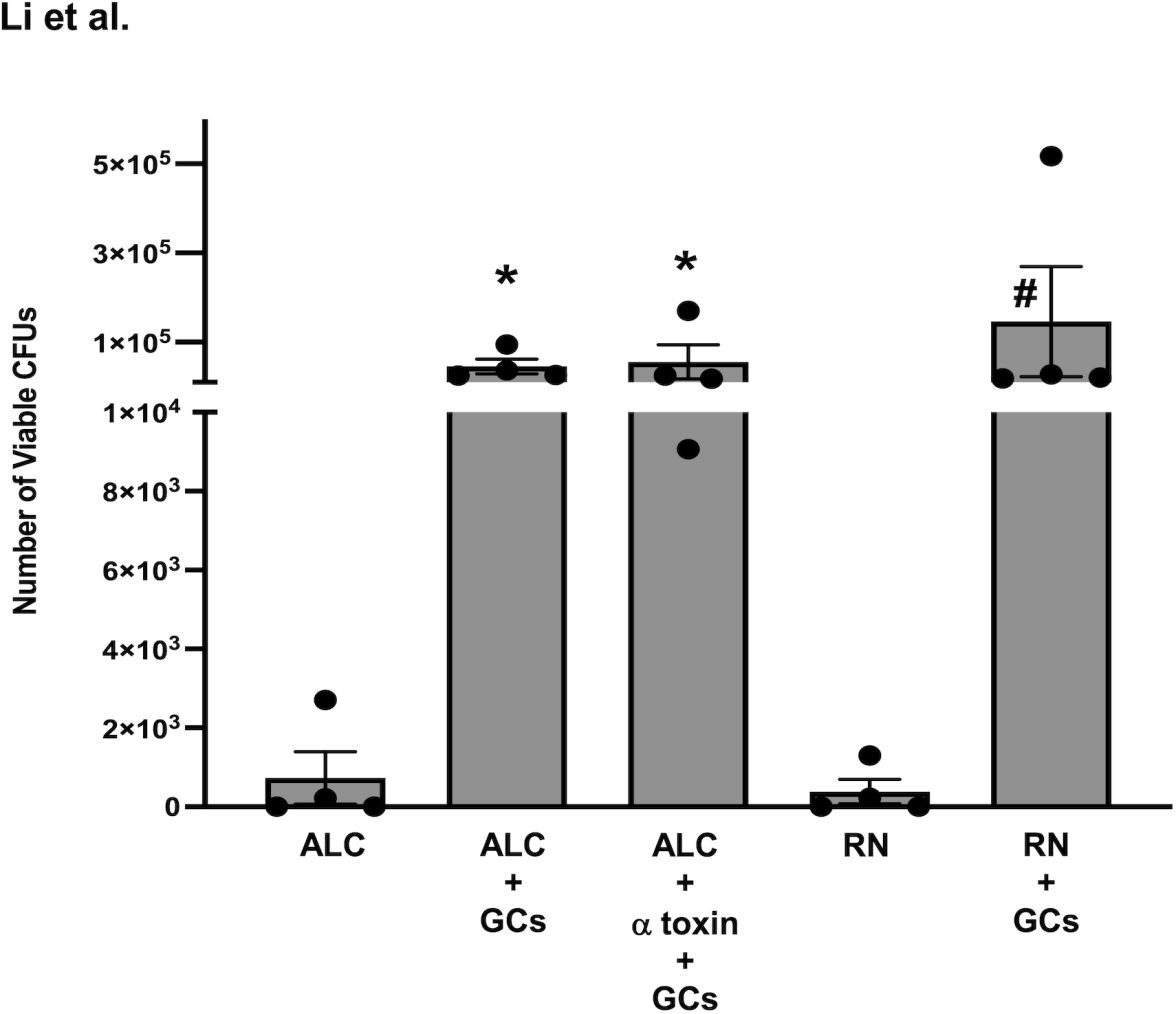
Internalization of *S. aureus* into human conjunctival goblet cells. Non-toxigenic ALC837 *S. aureus* (ALC) and toxigenic *S. aureus* RN6390 (RN) were incubated for 4 h (both at a multiplicity of infection (MOI) of 1:200) without or with human conjunctival goblet cells. Non-toxigenic ALC837 *S. aureus* plus α toxin was incubated for 4 h (at a MOI of 1:200) with human conjunctival goblet cells. For all groups, extracellular bacteria were killed with gentamicin for an additional 2 h. The goblet cells were lysed, serially diluted in 1X PBS, and plated on BHI-agar plates, and colonies were grown overnight at 37°C. Colony-forming units were counted for all conditions. Data are mean ± SEM from four independent experiments. *, significant difference from strain ALC837 alone; #, significant difference from strain RN6390 alone.

To answer this question, we first examined the internalization of the toxigenic *S. aureus* RN6390 and its α toxin mutant ALC837 in human conjunctival goblet cells. In this study, human conjunctival goblet cells were infected at an MOI of 1:200 with *S. aureus* RN6390, *S. aureus* ALC837, or *S. aureus* ALC837 plus α toxin (3 μg/ml) and incubated for 4 h, followed by gentamicin (200 μg/ml) for an additional 2 h (**Figure 6**). As a negative control, *S. aureus* RN6390 and *S. aureus* ALC837 were incubated for 4 h in the absence of cells and then treated for 2 h with gentamicin. In the negative controls, only 3.07 × 10^2^ ± 2.52 × 10^2^ and 5.85 × 10^2^ ± 5.32 × 10^2^ CFUs were detected in the *S. aureus* RN6390 (**Figure 6**, bar 4) and *S. aureus* ALC837 (bar 1) plates, respectively. These negative controls indicated the efficiency with which gentamicin kills extracellular bacteria. In contrast, the lysing of conjunctival goblet cells incubated with strains ALC837 (bar 2), ALC837 with α toxin (bar 3), and RN6390 (bar 5) for 4 h, followed by a 2-h treatment with gentamicin to kill all extracellular bacteria, revealed 4.57 × 10^4^ ± 1.65 × 10^4^, 5.56 × 10^4^ ± 3.83 × 10^4^ and 1.46 × 10^5^ ± 1.23 × 10^5^ CFUs/ml internalized bacteria, respectively. Thus, both toxigenic and non-toxigenic *S. aureus* are efficiently internalized in human conjunctival goblet cells, and the presence or absence of α toxin has no effect on the internalization of *S. aureus.*

### 3.6 Intracellular interaction of TLR2 and α toxin signaling pathways is needed for the induction of pro-IL-1 β synthesis and secretion of mature IL-1β in human conjunctival goblet cells

**Figure 7:**
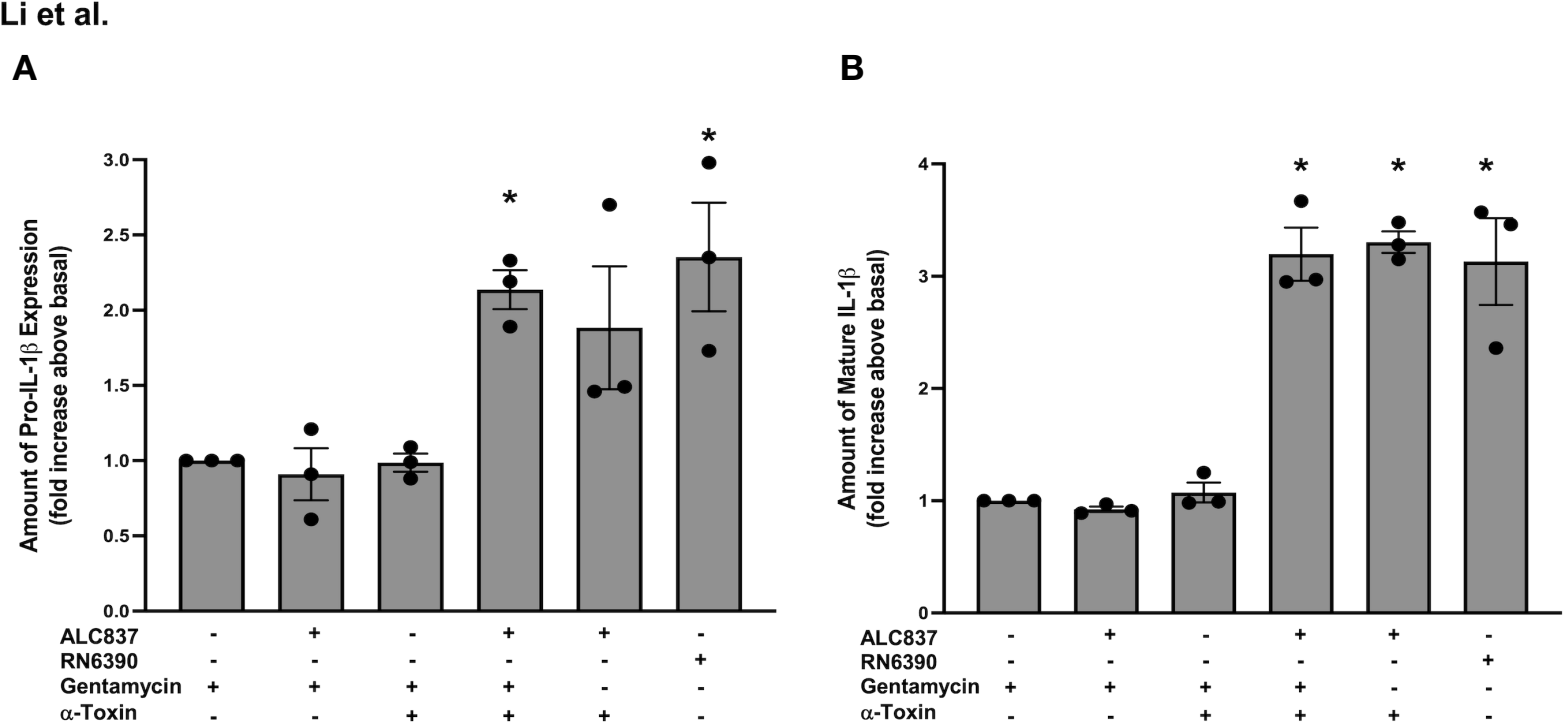
Intracellular interaction of TLR2 and α toxin pathways is needed for an increase in pro-IL1β synthesis and mature IL-1β secretion. Cultured human conjunctival goblet cells were treated with 200 μg/ml gentamicin, *S. aureus* ALC837 (multiplicity of infection, 20) followed by gentamycin, gentamicin followed by 1 μg/ml α toxin, *S. aureus* ALC837 followed by gentamycin and then α toxin, *S. aureus* ALC837 followed by α toxin, or *S. aureus* RN6390 for 4 h. The amount of pro-IL1β was measured by Western blot. The blots were scanned, and the amount of pro-IL1β is plotted in **(A)**. The amount of secreted mature IL-1β was measured by ELISA and is shown in **(B)**. Data are mean ± SEM from three independent experiments. *, significant difference from basal.

After confirming that (i) toxigenic and non-toxigenic *S. aureus* are both efficiently internalized in human conjunctival goblet cells and (ii) the presence or absence of α toxin has no effect on the internalization of *S. aureus*, we next sought to determine if α toxin facilitates the interaction between the internalized bacteria and intracellular TLR2 as well as the activation of the inflammasome (see **Figure 5A**). The addition of *S. aureus* ALC837 alone for 4 h followed by gentamycin for 2 h to kill extracellular bacteria and then buffer for 30 min did not increase the pro-IL-1β amount (**Supplementary Figure S6**, lane 2; **Figure 7A**, lane 2) or mature IL-1β secretion (**Figure 7B**, lane 2) compared to basal which was gentamycin only (**Supplementary Figure S6**, lane 1; **Figures 7A, B**, lane 1). The addition of α toxin alone (no bacteria) for 30 min following a 4-h incubation of buffer and a 2-h incubation with gentamicin also did not increase the level of pro-IL-1β (**Supplementary Figure S6**, lane 3) or secretion of mature IL-1β (**Figures 7A, B**, lane 3). Thus, neither internalized α toxin-deficient bacteria alone nor α toxin alone was effective at inducing the synthesis of pro-IL-1β or secretion of mature IL-1β. The addition of *S. aureus* ALC837 alone for 4 h followed by gentamicin to kill all extracellular bacteria and then α toxin for 30 min increased the level of both the pro-IL-1β amount to 2.1 ± 0.1-fold and the mature IL-1β secretion to 3.2 ± 0.2-fold over basal (**Supplementary Figure S6**, lane 4; **Figures 7A, B**, lane 4). As a positive control, the addition of α toxin-deficient bacteria for 4 h followed by buffer for 2 h (the extracellular bacteria remain alive) and then α toxin for 30 min also increased the pro-IL-1β amount and mature IL-1β secretion, but not significantly, unlike when extracellular bacteria were killed (**Supplementary Figure S6**, lane 5; **Figures 7A, B**, lane 5). A second positive control, the addition of toxigenic *S. aureus* RN6390, increased the pro-IL-1β amount and mature IL-1β secretion to the same level as when strain ALC837 was added, extracellular bacteria were killed, and then α toxin was added (**Supplementary Figure S6**, lane 6; **Figures 7A, B**, lane 6).

Our results indicate that α toxin-deficient *S. aureus* ALC837 is internalized, but the pathway to synthesize pro-IL-1β is not activated until the addition of α toxin, allowing the internalized bacteria to interact with internalized TLR2 and TLR1. The effect of toxigenic *S. aureus* RN6390 is the same as α toxin-deficient *S. aureus* ALC837 plus α toxin. Thus, α toxin not only activates the NLRP3/caspase-1 pathway but also interacts with the TLR2/NFκB pathway. Both signals, activation of TLR2 and activation of NLRP3/caspase-1, are needed for the production of pro-IL1β and secretion of mature IL-1β and are dependent upon α toxin.

## 4 Discussion

In the current paradigm for NLRP3 inflammasome activation, two distinct signals were thought to be required for the activation of the NLRP3 inflammasome and secretion of mature IL-1β (Franchi et al., 2009, Kelley et al., 2019). The first signal, the priming signal, provided by bacterial pathogen-associated molecular patterns (PAMPs) or endogenous danger-associated molecular patterns activates NF-κB via TLRs, resulting in the upregulation of NLRP3 and pro-IL-1β. The second signal, the activation signal, is provided by a variety of stimuli, including extracellular ATP and pore-forming toxins, and a decrease in intracellular [K^+^] induces inflammasome formation and the activation of caspase-1. The activated caspase-1 then cleaves the synthesized pro-IL-1β to release mature IL-1β. In contrast, our results presented herein suggest an alternative mechanism with one signal, the permeabilization of cellular and intracellular membranes by α toxin. Upon infection of conjunctival goblet cells with toxigenic *S. aureus*, some bacteria remain extracellular, while some bacteria are internalized and appear to be localized within an intracellular compartment. TLR2 and TLR1 are also localized intracellularly but appear to be in a different compartment from the bacteria. Thus, the bacteria and its associated PAMPs are not able to interact with and activate the TLRs. The activation of TLR2 and, to some extent, TLR1 seems to be dependent upon the release of α toxin from the internalized toxigenic *S. aureus* and the subsequent formation of pores in the membranes of the different intracellular compartments, allowing the interaction of the bacterial PAMPs with the TLRs. TLR activation increases the synthesis of both pro-IL1β and the components of the inflammasome. At the same time, bacterial α toxin from the extracellular bacteria permeabilizes the plasma membrane, resulting in an efflux of cytosolic [K^+^] and influx of [Ca^2+^] that trigger the formation and activation of the NLRP3 inflammasome in which caspase-1 cleaves synthesized pro-IL1β and to mature IL-1β and is released. The intracellular location of TLR2 is consistent with the experiments of Ueta et al. (Ueta et al., 2004) who found that TLR2 is not expressed on the surface of ocular mucosal epithelial cells. In addition, TLR2 is also intracellularly localized in the endosomes of monocytes and macrophages (Brandt et al., 2013).

The requirement of pore formation for TLR signaling is supported by our data demonstrating that the mutant *S. aureus* ACL837, that is only missing the α toxin, fails to trigger TLR activation and the upregulation of pro-IL1β. These results also suggest that both bacterial PAMPs and α toxin are needed for the induction of pro-IL1β synthesis and mature IL-1β secretion. This conclusion is also supported by our results demonstrating as follows: (i) in the absence of TLR2 activation, by knocking down TLR2, toxigenic *S. aureus* is unable to induce pro-IL1β synthesis nor trigger caspase-1 activation and the secretion of mature IL-1β and (ii) the addition of α toxin alone (no bacterial PAMPs) fails to induce pro-IL-1β synthesis and is unable to activate caspase-1 or stimulate mature IL-1β secretion. This finding is in contrast to that of Hruz (Hruz et al., 2009) who found that TLR2- and TLR4-deficient macrophages can still produce mature IL-1β in response to toxigenic *S. aureus* (Hruz et al., 2009). In the macrophages, α toxin facilitates the entry of bacteria into the cytoplasm where the bacteria interact with NOD2. Our finding is also different from that of Nilsen et al. (Nilsen et al., 2008) who found, in monocytes, that lipoteichoic acid that mimics gram-positive PAMPs activated NFκB without being internalized in spite of the fact that TLR2 can be rapidly internalized. In contrast to these two studies, our data show that, in the absence of TLR2, goblet cells do not respond to toxigenic *S. aureus*. Thus, α toxin appears to have two actions in conjunctival goblet cells, namely: (i) to permeabilize intracellular compartments to allow the interaction of bacterial PAMPs with TLRs and the induction of pro-IL1β and (ii) to decrease intracellular [K^+^], resulting in the activation of the NRLP3 inflammasome. While TLR2 and TLR1 are required for the activation of the NRLP3 inflammasome, they seem to remain inactive in the absence of α toxin.

The mechanism of NLRP3 inflammasome activation by TLRs and pore formation induced by extracellular ATP or a pore-forming toxin has been predominantly studied in immune cells such as macrophages and neutrophils using animal models of disease or cultured cells such as THP-1 cells (Netea et al., 2008; Babelova et al., 2009; Harder et al., 2009; Netea et al., 2009; Cho et al., 2012). The mechanism of NLRP3 activation and mature IL-1β secretion, however, differs between cells and tissues. Macrophages require two signals, such as bacterial products to prime and ATP or crystal-induced lysosomal damage to activate the NRLP3 inflammasome (Bauernfeind et al., 2009). In skin keratinocytes, serum amyloid A alone triggered inflammasome activation and IL-1β secretion through the activation of TLR2 and TLR4 (Yu et al., 2015). The use of a pore-forming agent appeared not to be necessary, but serum amyloid A activates reactive oxygen species (ROS), and it was demonstrated that the activation of the NLRP3 inflammasome by serum amyloid A was dependent upon ROS generation as a second signal. In contrast, not all cell types, when stimulated by bacteria or other TLR-dependent ligands, require two different signals to activate the NRLP3 inflammasome—for example, monocytes require only stimulation of TLR ligands to secrete mature IL-1β via NLRP3 activation (Netea et al., 2008). However, in other cell types, such as airway smooth muscle cells, incubation with the bacterial PAMP, Pam3CSK4, results in the activation of NF-κB via TLR2 and upregulation of pro-IL-1β but did not result in the secretion of mature IL-1β. This finding demonstrates that the NLRP3 inflammasome was not activated upon TLR2 ligation alone (Hirota et al., 2013). Thus, the mechanism of NRLP3 activation and secretion of mature IL-1β appears to be cell-specific.

The strain of toxigenic *S. aureus* RN6390 used in the present study harbors genes for several toxins, including α, β, and γ (Peng et al., 1988). We demonstrated herein that the deletion of only the α toxin removed the ability of *S. aureus* to induce the synthesis of pro-IL1β, activate the NLRP3 inflammasome, and stimulate the secretion of mature IL-1β. Adding the α toxin back restored the response, which is in agreement with previous work demonstrating that α toxin is critical for the activation of the NLRP3 inflammasome in human and mouse monocytic cells in response to toxigenic bacteria (Craven et al., 2009). Multiple cell types are sensitive to α toxin, including erythrocytes, platelets, monocytes, neutrophils, T cells, pneumocytes (surface epithelial cells of lung alveoli), epidermal keratinocytes, and endothelial cells (Berube and Bubeck Wardenburg, 2013). The epithelial goblet cells of the conjunctiva can now be added to this list. Similarly to monocytes, the goblet cells also respond to the combination of α toxin and *S. aureus*, which together with TLR recognition have a greater effect on NLRP3 activation and mature IL-β secretion than α toxin alone (Craven et al., 2009).

In conjunctival goblet cells, only toxigenic *S. aureus*, but not non-toxigenic *S. aureus* (missing α, β, and γ toxins) nor commensal *S. epidermidis*, activates the NLRP3 inflammasome to produce mature IL-1β (Li et al., 2016). We suggest a mechanism by which goblet cells distinguish between these toxigenic, non-toxigenic, and commensal bacteria even when the different bacteria express the same PAMPs. While we demonstrate that α toxin is not needed for bacterial internalization, our data indicate that α toxin present in the toxigenic bacteria is important to facilitate the interaction of intracellular bacteria and their PAMPs with intracellular TLRs to initiate the synthesis of pro-IL-1β and activation of the NLRP3 inflammasome.

Conjunctival goblet cells constitutively express pro IL-1β and all components of the NLRP3 inflammasome (McGilligan et al., 2013). This finding is in agreement with the constitutive presence of NLRP3 in wet-surfaced mucosa, including the conjunctiva (Kummer et al., 2007), in contrast to inflammatory cells in which these components need to be first synthesized. The constitutive presence of NLRP3 in the conjunctival goblet cells allows the rapid protective response of these cells to the environment. In the present experiments, we did not demonstrate a causal relationship between TLR2 and NRLP3. In the future experiments in which TLR2 is increased and activation of NRLP3 is measured, it would be critical. In addition, we did not determine if a decrease or an increase in TLR2 affected the viability of the toxigenic *S. aureus* bacteria. Future experiments determining this would strengthen our present results.

We conclude that α toxin from toxigenic *S. aureus* is one of several mechanisms that can activate the NRLP3 inflammasome. We conclude that it triggers two separate mechanisms required for the activation of the NLRP3 inflammasome and secretion of mature IL-1β. In the first mechanism, α toxin secreted from internalized *S. aureus* produces a pore, allowing the internalized bacteria and associated PAMPs to interact with intracellular TLR2 and, to a lesser extent, TLR1. In the second mechanism, α toxin forms a pore in the plasma membrane, leading to an efflux of cytosolic K^+^ and influx of Ca^2+^. The activation of these two mechanisms together triggers increased synthesis of pro-IL-1β and the components of the NLRP3 inflammasome as well as activation of the NLRP3 inflammasome and secretion of mature IL-1β, with the ultimate goal of eliminating the bacterial pathogens and protecting the ocular surface.

## Data availability statement

The raw data supporting the conclusions of this article will be made available by the authors without undue reservation.

## Ethics statement

The studies involving human tissues were approved by Schepens Eye Research Institutional Review Board and were not considered to be human studies research. The studies were conducted in accordance with the local legislation and institutional requirements. The tissue was purchased from Eye Banks who procured it from cadavers with patient's consent.

## Author contributions

DD: Conceptualization, Funding acquisition, Project administration, Resources, Supervision, Writing – original draft, Writing – review & editing. DL: Formal Analysis, Investigation, Writing – original draft. RH: Formal Analysis, Investigation, Methodology, Writing – review & editing. MA: Formal Analysis, Investigation, Writing – original draft. JB: Formal Analysis, Investigation, Methodology, Writing – review & editing. PB: Methodology, Supervision, Writing – review & editing. MG: Resources, Supervision, Writing – review & editing. MG-K: Conceptualization, Funding acquisition, Resources, Supervision, Writing – review & editing.

## Supplementary material

Supplementary Figure 1 TLR2 plays a role in *S. aureus* RN6390-induced activation of NLRP3 inflammasome and production of pro-IL-1β. Cultured human conjunctival goblet cells were treated with 50 or 100 nM siRNA against TLR2 or scrambled siRNA (sc-siRNA). Western blot analysis showing depletion of TLR2 is shown in **(A)**. The blot is from one experiment. Goblet cells were incubated with *S. aureus* RN6390 (multiplicity of infection, 20) in the presence or absence of 50 nM TLR2 siRNA or sc-siRNA and Western blot analysis was performed for TLR2 and is shown in **(B)**. The amount of pro-ILβ was determined by Western blot and is shown in **(C)**. The amount of caspase 1 was determined by FLICA assay, and representative micrographs are shown in **(D)**. The blots and FLICA are shown as a single result from three independent experiments.

Supplementary Figure 2 TLR1 plays a role in *S. aureus* RN6390-induced production of pro-IL-1β. Cultured human conjunctival goblet cells were treated with 50 nM siRNA against TLR1 or scrambled siRNA (sc-siRNA). Western blot analysis was performed for pro-ILβ, and a representative blot from three independent experiments is shown.

Supplementary Figure 3 TLR6 plays a role in *S. aureus* RN6390-induced activation of NRLP3 inflammasome and production of pro-IL-1β. Cultured human conjunctival goblet cells were treated with 25, 50, or 100 nM siRNA against TLR6 or scrambled siRNA (sc-siRNA). Western blot analysis showing the depletion of TLR6 is shown in **(A)**. The blot is from one experiment. Goblet cells were incubated with *S. aureus* RN6390 (multiplicity of infection, 20) for 4 h in the presence or absence of 100 nM TLR6 siRNA or sc-siRNA. Western blot analysis was performed for TLR6, and the results are shown in **(B)**. The amount of pro-ILβ was also determined by Western blot. A representative blot is shown in **(C)**. The amount of caspase 1 was determined by FLICA. Representative micrographs are shown in **(D)**. The blots and FLICA are shown as a single result from three independent experiments except where indicated.

Supplementary Figure 4
*S. aureus* RN6390 requires α toxin to induce the production of pro-IL1β. Cultured human conjunctival goblet cells were treated with 1 μg/ml α toxin, *S. aureus* RN6390, or *S. aureus* ALC837 (multiplicity of infection, 20) for 4 h. The amount of pro-IL1β was measured by Western blot, and a representative blot is shown. The blot is shown as a single result from three independent experiments.

Supplementary Figure 5
*S. aureus* ALC837 requires α toxin to induce the production of pro-IL1β. Cultured human conjunctival goblet cells were treated with *S. aureus* ALC837 (multiplicity of infection, 20), 1 μg/ml α toxin, and *S. aureus* RN6390 or ALC837 plus α toxin for 4 h. The amount of pro-IL1β was measured by Western blot, and a representative blot is shown. The blot is shown as a single result from three independent experiments.

Supplementary Figure 6 Intracellular interaction of TLR2 and α toxin pathways is required for an increase of pro-IL1 β synthesis. Cultured human conjunctival goblet cells were treated with 200 μg/ml gentamicin, *S. aureus* ALC837 (multiplicity of infection, 20) followed by gentamycin, gentamicin followed by 1 μg/ml α toxin, *S. aureus* ALC837 followed by gentamycin and then α toxin, *S. aureus* ALC837 followed by α toxin, or *S. aureus* RN6390 for 4 h. The amount of pro-IL1β was measured by Western blot, and a representative blot from three independent experiments is shown.
