# Supplementary figures and images for: Staphylococcus aureus activates NRLP3-dependent IL-1β secretion from human conjunctival goblet cells using α toxin and toll-like receptors 2 and 1
Source: Front Cell Infect Microbiol. 2023 Nov 27;13:1265471. doi: 10.3389/fcimb.2023.1265471 (PMC10711068; doi:10.3389/fcimb.2023.1265471)

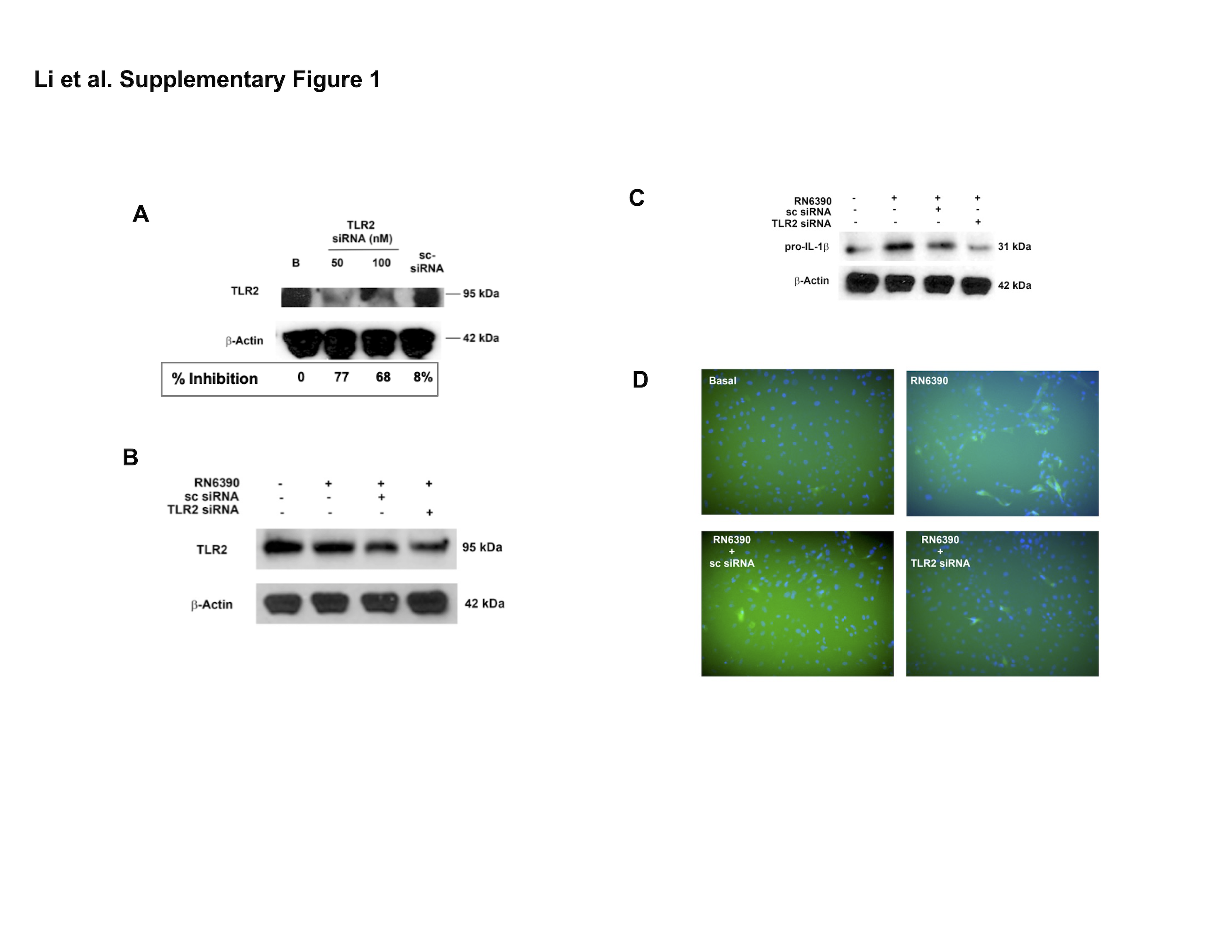

Supplement: Supplementary Figure 1 — TLR2 plays a role in S. aureus RN6390-induced activation of NLRP3 inflammasome and production of pro-IL-1β. Cultured human conjunctival goblet cells were treated with 50 or 100 nM siRNA against TLR2 or scrambled siRNA (sc-siRNA). Western blot analysis showing depletion of TLR2 is shown in (A). The blot is from one experiment. Goblet cells were incubated with S. aureus RN6390 (multiplicity of infection, 20) in the presence or absence of 50 nM TLR2 siRNA or sc-siRNA and Western blot analysis was performed for TLR2 and is shown in (B). The amount of pro-ILβ was determined by Western blot and is shown in (C). The amount of caspase 1 was determined by FLICA assay, and representative micrographs are shown in (D). The blots and FLICA are shown as a single result from three independent experiments. [file Image_1.tiff]

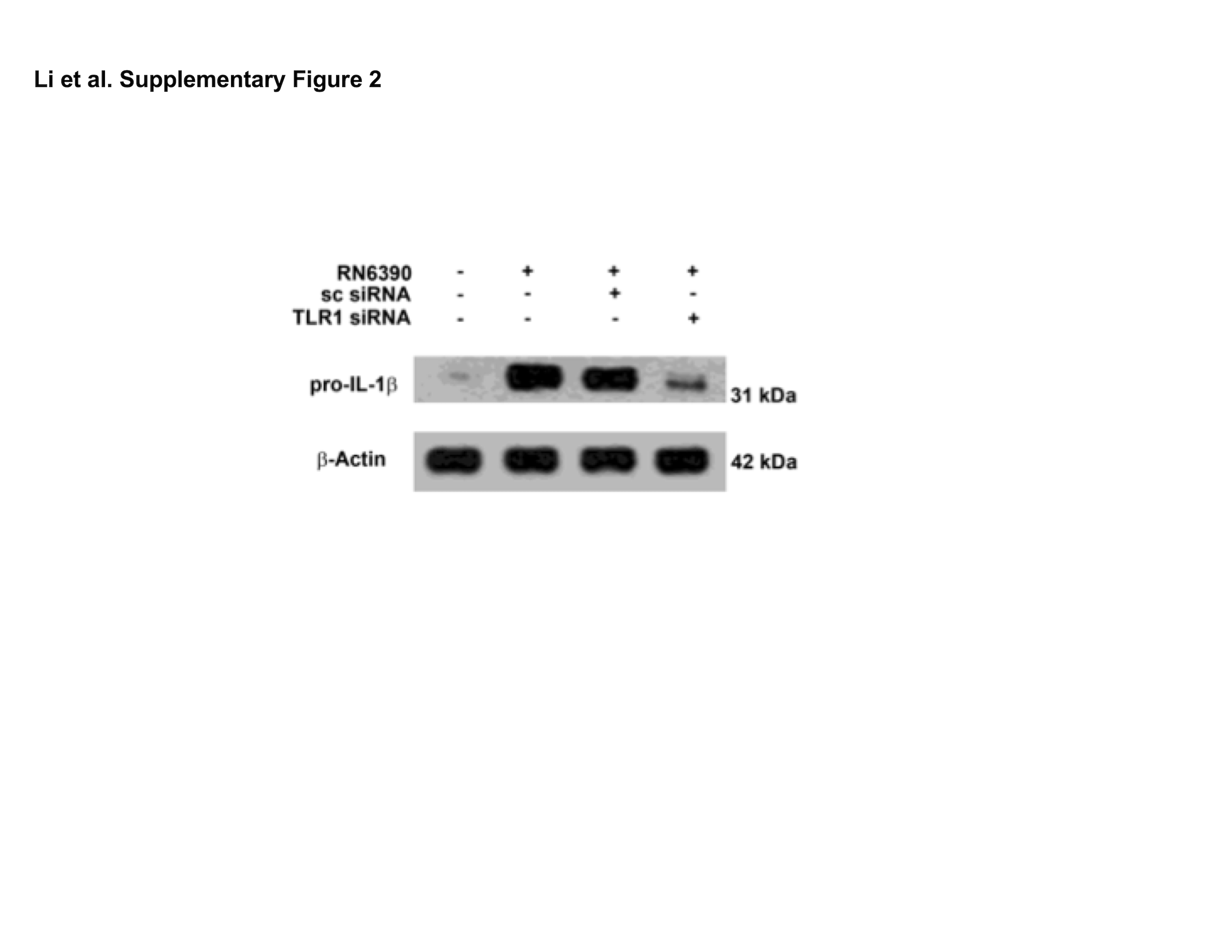

Supplement: Supplementary Figure 2 — TLR1 plays a role in S. aureus RN6390-induced production of pro-IL-1β. Cultured human conjunctival goblet cells were treated with 50 nM siRNA against TLR1 or scrambled siRNA (sc-siRNA). Western blot analysis was performed for pro-ILβ, and a representative blot from three independent experiments is shown. [file Image_2.tiff]

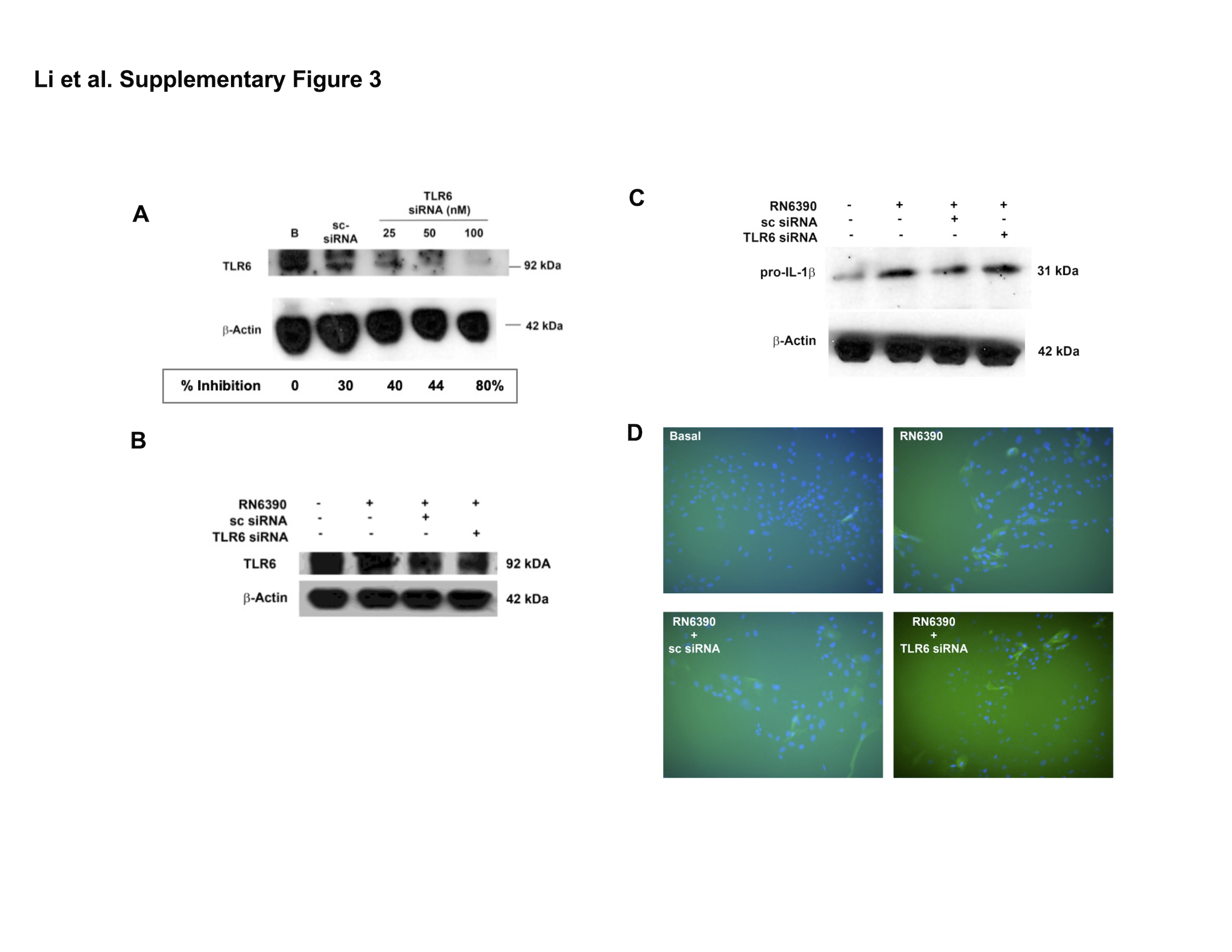

Supplement: Supplementary Figure 3 — TLR6 plays a role in S. aureus RN6390-induced activation of NRLP3 inflammasome and production of pro-IL-1β. Cultured human conjunctival goblet cells were treated with 25, 50, or 100 nM siRNA against TLR6 or scrambled siRNA (sc-siRNA). Western blot analysis showing the depletion of TLR6 is shown in (A). The blot is from one experiment. Goblet cells were incubated with S. aureus RN6390 (multiplicity of infection, 20) for 4 h in the presence or absence of 100 nM TLR6 siRNA or sc-siRNA. Western blot analysis was performed for TLR6, and the results are shown in (B). The amount of pro-ILβ was also determined by Western blot. A representative blot is shown in (C). The amount of caspase 1 was determined by FLICA. Representative micrographs are shown in (D). The blots and FLICA are shown as a single result from three independent experiments except where indicated. [file Image_3.tiff]

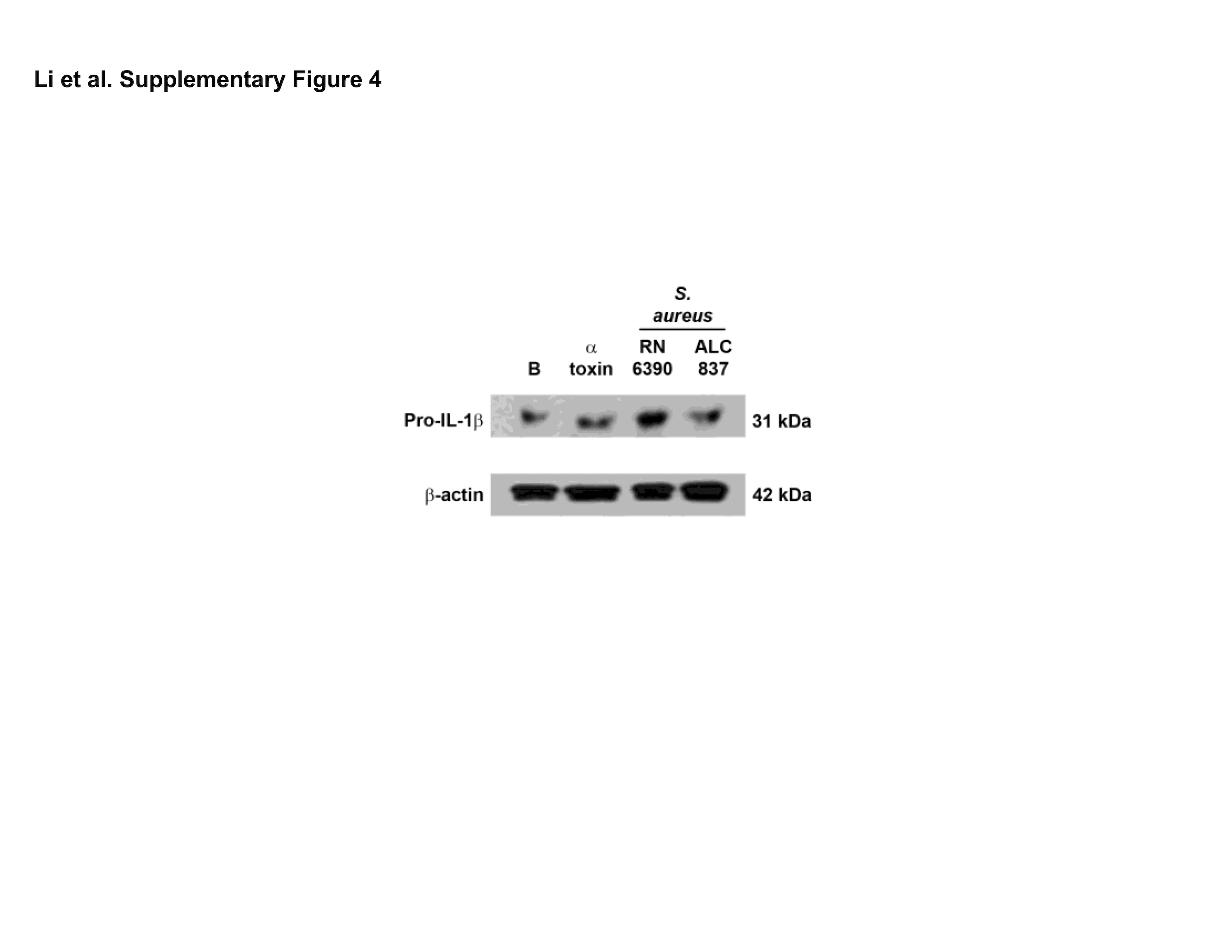

Supplement: Supplementary Figure 4 — S. aureus RN6390 requires α toxin to induce the production of pro-IL1β. Cultured human conjunctival goblet cells were treated with 1 μg/ml α toxin, S. aureus RN6390, or S. aureus ALC837 (multiplicity of infection, 20) for 4 h. The amount of pro-IL1β was measured by Western blot, and a representative blot is shown. The blot is shown as a single result from three independent experiments. [file Image_4.tiff]

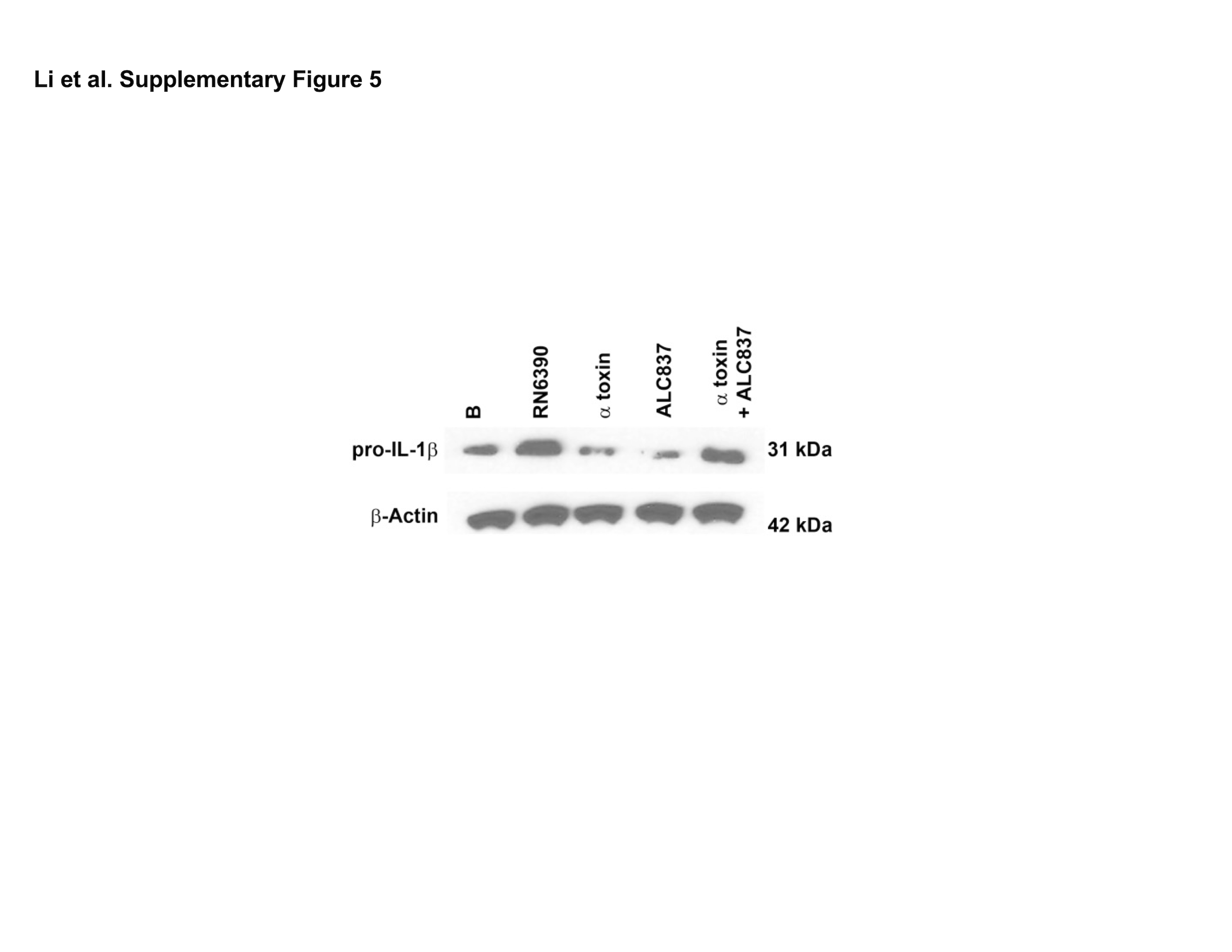

Supplement: Supplementary Figure 5 — S. aureus ALC837 requires α toxin to induce the production of pro-IL1β. Cultured human conjunctival goblet cells were treated with S. aureus ALC837 (multiplicity of infection, 20), 1 μg/ml α toxin, and S. aureus RN6390 or ALC837 plus α toxin for 4 h. The amount of pro-IL1β was measured by Western blot, and a representative blot is shown. The blot is shown as a single result from three independent experiments. [file Image_5.tiff]

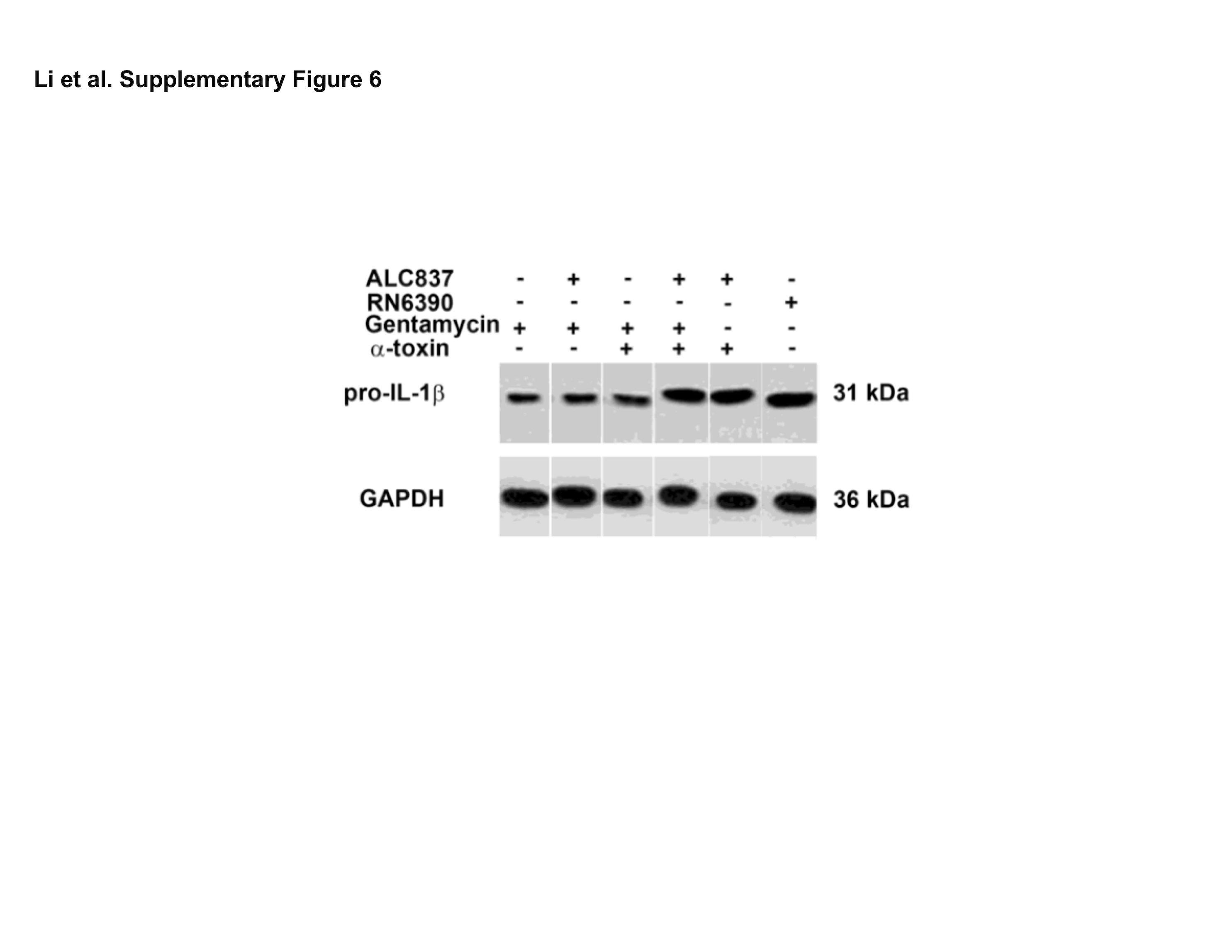

Supplement: Supplementary Figure 6 — Intracellular interaction of TLR2 and α toxin pathways is required for an increase of pro-IL1 β synthesis. Cultured human conjunctival goblet cells were treated with 200 μg/ml gentamicin, S. aureus ALC837 (multiplicity of infection, 20) followed by gentamycin, gentamicin followed by 1 μg/ml α toxin, S. aureus ALC837 followed by gentamycin and then α toxin, S. aureus ALC837 followed by α toxin, or S. aureus RN6390 for 4 h. The amount of pro-IL1β was measured by Western blot, and a representative blot from three independent experiments is shown. [file Image_6.tiff]
